# Heterogeneous staining: a tool for studies of how fluorescent dyes affect the physical properties of DNA

**DOI:** 10.1093/nar/gkt755

**Published:** 2013-08-23

**Authors:** Lena Nyberg, Fredrik Persson, Björn Åkerman, Fredrik Westerlund

**Affiliations:** ^1^Department of Chemical and Biological Engineering, Chalmers University of Technology, Gothenburg, Sweden and ^2^Department for Cell and Molecular Biology, Uppsala University, Uppsala, Sweden

## Abstract

The commonly used fluorescent dye YOYO-1 (YOYO) has, using bulk techniques, been demonstrated to stain DNA heterogeneously at substoichiometric concentrations. We here, using nanofluidic channels and fluorescence microscopy, investigate the heterogeneous staining on the single DNA molecule level and demonstrate that the dye distribution is continuous. The equilibration of YOYO on DNA is extremely slow but can be accelerated by increasing the ionic strength and/or the temperature. Furthermore, we demonstrate how to use the heterogeneous staining as a tool for detailed and time-efficient studies of how fluorescent dyes affect the physical properties of DNA. We show that the relative increase in extension of DNA with increasing amount of YOYO bound is higher at low ionic strengths and also extrapolate the extension of native DNA. Our study reveals important information on how YOYO affects the physical properties of DNA, but it also has broader applications. First, it reveals how cationic intercalators, such as potential DNA drugs, affect DNA under strong confinement. Second, the strategy of using heterogeneous staining is of general use for single molecule studies of DNA interacting with proteins or ligands.

## INTRODUCTION

Single molecule techniques have become important tools for studying interactions between DNA and DNA-binding ligands or proteins (1,2). One main advantage, compared with standard bulk techniques, is the possibility to detect and characterize sample heterogeneity in a straightforward fashion. Commonly used techniques for studying single DNA molecules and their interactions include optical (3) and magnetic (4) tweezers where the DNA is generally stretched beyond its contour length with forces in the piconewton regime.

The use of nanofluidic channels, combined with fluorescence microscopy, has emerged as a powerful tool for studies of single DNA molecules (5,6) with applications ranging from fundamental polymer physics (7,8) to DNA/protein interactions (9–11) and optical sequence mapping (12,13). A DNA molecule enclosed in a channel with two dimensions smaller than its radius of gyration will spontaneously stretch out along the channel with an extension that is generally 30–70% of the contour length of the DNA. The DNA is thus not fully stretched in the nanochannel but rather fluctuates around a mean extension that mainly depends on the channel dimensions and the properties of the surrounding medium, e.g. ionic strength (14–16). As the DNA is not fully stretched out, intramolecular interactions are important and can be studied. Contrary to several other single DNA molecule techniques, the DNA does not have to be attached to any surfaces or ‘handles’. Any DNA of sufficient length, including circular DNA, can thus be investigated with high throughput and potentially in an automated fashion.

**Figure 1.**
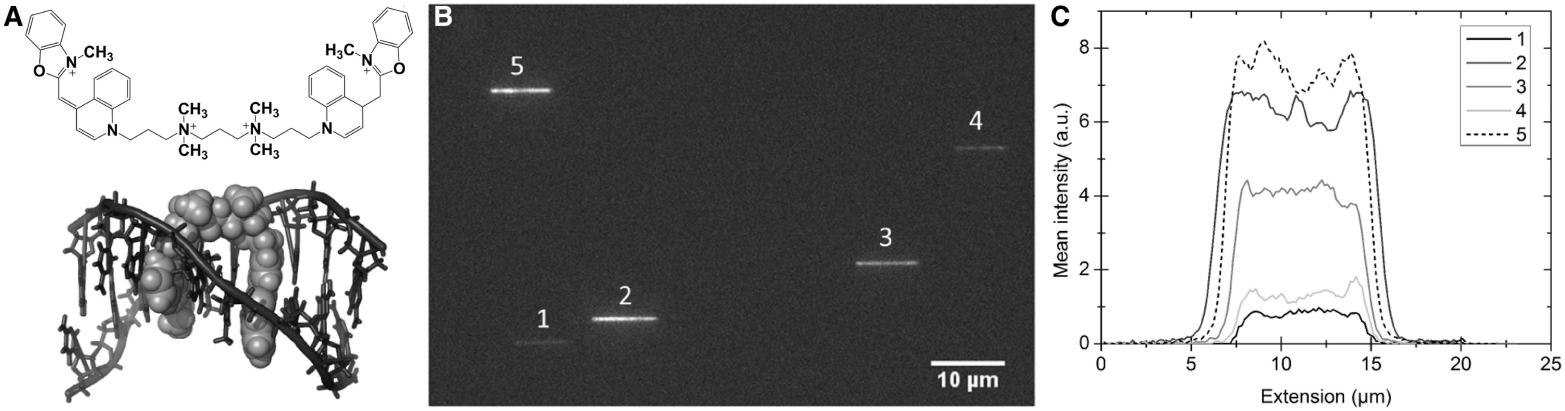
(**A**) Chemical structure of YOYO-1. (**B**) NMR structure of the bis-intercalating YOYO-analogue TOTO-1 bound to DNA, visualized with PyMOL (28). (**C**) Fluorescence microscopy image of five λ-DNA molecules in the same field of view trapped in nanochannels with the dimensions 100 × 150 nm^2^. (**D**) Corresponding intensity traces for the five λ-DNA molecules in (C) averaged over 200 consecutive images for each molecule at a dye:bp ratio of 1:10 in 0.5× TBE buffer.

As DNA itself is non-fluorescent, labeling is necessary to study DNA by fluorescence microscopy. *Bis*-intercalating cyanine dyes such as YOYO-1 (YOYO, Figure 1A) and TOTO-1 were introduced in 1992 (17) and are by far the most commonly used fluorophores for single molecule studies of double-stranded DNA in general and for nanofluidic applications in particular (5,6). One of the main reasons for the popularity of dimeric cyanine dyes is that they exhibit an ∼1000-fold enhancement in emission on binding to double-stranded DNA, greatly reducing problems with background fluorescence from unbound dye (17). YOYO carries four positive charges and binds, like its analogue TOTO-1, by *bis*-intercalating its two chromophore units into the DNA (Figure 1B) (18). On binding, YOYO alters the overall charge of the DNA–YOYO complex as well as the physical properties of DNA such as contour- and persistence length and helical pitch (19–22). As an example, the effective electrophoretic charge of the DNA–YOYO complex decreases with ∼15%, compared with native DNA, at a dye load of one YOYO molecule every 10 bp (23). No detailed studies have, to our knowledge, yet been performed to conclude how YOYO affects the extension of nanoconfined DNA, but there are suggestions that YOYO affects DNA extension in other ways than the mere increase in contour length due to intercalation (7,8).

In 1995, it was discovered that electrophoresis of a DNA sample stained with YOYO at substoichiometric concentrations (i.e. dye:basepair ratios less than the nearest neighbor exclusion value of one YOYO molecule every 4 bp, 1:4), yields two gel bands, suggesting that the sample consists of two distinct populations with different electrophoretic properties, most likely due to different amounts of dye bound (24). It was concluded that the slower migrating band corresponded to DNA fully covered with YOYO, while the other band contained DNA molecules with less dye bound. For the two bands to merge into one, the sample had to be heated at 50°C for several hours, suggesting that the equilibration of YOYO on DNA is extremely slow. Slow on- and off-rates for YOYO have also been demonstrated on the single DNA molecule level using optical tweezers (25–27).

We here demonstrate, by studying single DNA molecules in nanochannels, that YOYO does stain DNA heterogeneously at substoichiometric concentrations. However, in contradiction to the electrophoresis study (24), we observe a continuous variation in staining rather than two distinct fractions. The equilibration of YOYO on DNA is much faster at high ionic strengths, suggesting that the slow equilibration is a result of the strong electrostatic attraction between the cationic dye and the negatively charged DNA. We also demonstrate how the heterogeneous staining can be used as a tool to study how YOYO affects the physical properties of nanoconfined DNA, from almost naked to fully saturated with dye, in a single experiment. Interestingly, the increase in extension of the DNA with increasing YOYO loading is more pronounced at low ionic strength. Furthermore, the assay allows us to extrapolate information about nanoconfined DNA without any dye bound. As an example, we demonstrate how the extension of nanoconfined naked DNA varies with ionic strength.

## MATERIALS AND METHODS

All DNA samples were mixed in TBE buffer (Medicago, 10 x TBE tablets) diluted in milli-Q water to the desired ionic strength. The oxygen scavenger β-mercaptoethanol (Sigma-Aldrich) was added to the buffer (3% v/v) to suppress photonicking of the DNA. YOYO-1 was purchased from Invitrogen and DNA from phage lambda (λ-DNA, 48.5 kb) was purchased from New England Biolabs.

The nanofluidic chips were fabricated in fused silica according to methods described elsewhere (6), with a cross-section of ∼100 × 150 nm^2^ and a length of ∼500 µm. All experiments were conducted in a nanofluidic chip consisting of pairs of microchannels that are spanned by nanochannels. The DNA sample solution was loaded into one of the microchannels of the chip and transferred to the nanochannel array by pressure. By applying pressure over two connected microchannels, the DNA was subsequently injected into the nanochannels. The DNA was imaged using an epi-fluorescence microscope (Zeiss AxioObserver.Z1) equipped with a Photometrics Evolve EMCCD camera and a 100× oil immersion TIRF objective (NA = 1.46) from Zeiss. Stacks of 200 images were recorded for each molecule, using the AxioVision software, with an exposure time of 100 ms per image at maximum speed, corresponding to approximately seven frames per second. Data analysis was performed with the freeware ImageJ (www.imagej.com) and a custom-written MatLab based software. For each molecule, kymographs (timetraces) were first extracted. Subsequently, each line in the kymograph—corresponding to one frame in the original time series—was fitted with a convolution of a box function and an error function (6). This gives the position and extension of the molecule in each frame that is used to align the kymograph as well as obtaining average intensities and fluctuations.

The fluorescence intensity was normalized to a distinct upper limit in fluorescence intensity identified from measurements. This upper limit is assumed to correspond to the fluorescence intensity of fully intercalated DNA, ∼1 YOYO molecule every 4 bp (21,22). Extrapolation of the extension of native DNA was obtained by a linear fit of the first eight binned values for each ionic strength. The straight lines in Figure 3B were calculated from the extrapolated extension of the native DNA, again assuming a maximal intercalation of one YOYO every 4 bp and adding the resulting increase in contour length of 0.51 nm per YOYO molecule (22), taking the relative extension of the native DNA compared with the full contour length into account.

## RESULTS AND DISCUSSION

### Heterogeneous staining of DNA by YOYO-1

The first goal of the present study was to characterize the heterogeneous staining of DNA by YOYO, previously reported in bulk measurements (24), on the single DNA molecule level. Figure 1C shows five λ-DNA molecules from a non-heated sample in the same field of view at a dye ratio that corresponds to an average of one YOYO molecule every 10 bp (hereafter denoted as a dye:bp ratio of 1:10). The marked intermolecular differences in mean intensity clearly demonstrate that YOYO stains DNA in a heterogeneous fashion. Already from a single image it can be observed that, rather than two distinct populations, there is a wide distribution of intensities, and hence dye loading, in the sample. This is confirmed in Figure 1D where intensity traces along each DNA molecule are shown. Furthermore, Figure 1D reveals that a higher emission intensity (i.e. a higher fraction of dye bound) results in a more extended DNA, as will be discussed later in the text.

**Figure 2.**
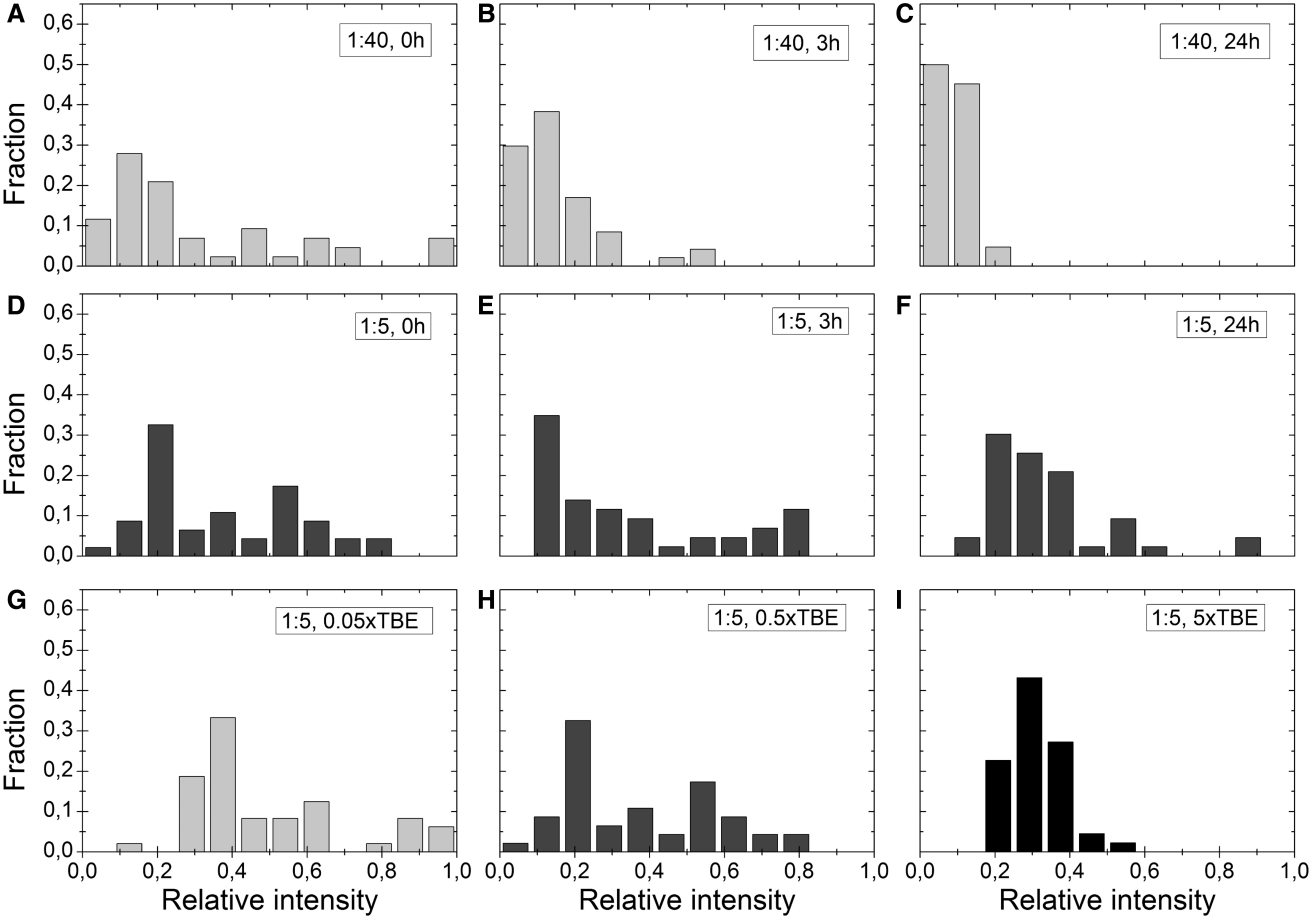
(**A–C**) Intensity fractions for λ-DNA molecules with a dye:bp ratio of 1:40 in 0.5× TBE. (**D–F**) Corresponding data for a dye:bp ratio of 1:5 in 0.5× TBE. (A) and (D) are samples that have not been heated (0 h), samples in (B) and (E) have been heated for 3 h at 50°C, and samples in (C) and (F) have been heated for 24 h at 50°C. (**G–I**) Intensity distributions at three different ionic strengths – 0.05×, 0.5× and 5× TBE – and a dye:bp ratio of 1:5. None of the samples were heated. Each set (A–I) comprises 40–50 molecules and corresponds to a single experiment. All experiments were repeated at least twice, and the results are in qualitative agreement. The graphs in D and H are identical; they are shown as duplicates to increase the readability of the figure.

To quantify the observations, we investigated the fluorescence intensity distributions in detail at dye:bp ratios that are commonly used in single molecule experiments. The sample solutions were mixed, divided into three aliquots, and heated for 0, 3 or 24 h at 50°C, respectively, to study the equilibration process. Figure 2A–C show how the intensity distribution changes with time and heating at a dye:bp ratio of 1:40. The intensity distribution in the sample at 0 h (Figure 2A) is highly scattered. Heating the sample results in a more homogenous intensity distribution but does not, even after 24 h of heating, result in a sample where all DNA molecules display the same level of fluorescence intensity. Furthermore, the mean intensity of the molecules analyzed in the non-heated sample (Figure 2A) is more than three times higher than for the sample that has been heated for 24 h (Figure 2C). We explain this decrease in integrated intensity of the sample as being due to that the dye distribution is much broader when the sample is not heated. In a non-heated sample with a low dye:bp ratio, not all molecules will have enough YOYO intercalated to be detected—we refer to these molecules as the ‘dark fraction’—and molecules with high fluorescence intensity will thus be over-represented. With time and heat, the dyes spread among all DNA molecules in the sample and the mean intensity per molecule will decrease. Figure 2D–F show the corresponding intensity distribution at a dye:bp ratio of 1:5. For the equilibrated sample (Figure 2F), it is evident that the average dye intensity is higher compared with a staining ratio of 1:40 (Figure 2C), as expected for a higher dye loading. This observation is however less obvious directly after mixing the sample (0 h, Figure 2A and D). We believe that at high dye loading, the dark fraction is much smaller. For the higher dye concentration (1:5), the mean intensity is similar for both the heated and non-heated samples, implying that all DNA molecules have enough intercalated dyes to be detected in the 0 h sample, but the intensity distribution is extremely wide, and thus the samples at 1:5 and 1:40 (Figure 2A and D) appear similar. Furthermore, at 1:5 the intensity does not even out with time as readily as for the lower dye concentration, even though the trend is similar. In conclusion, we find that non-heated samples of DNA stained with YOYO at very different dye:bp ratios may appear similar in dye distribution (Figure 2A and D) due to heterogeneous staining in conjunction with the detection limits of the camera.

To understand the origin of the heterogeneous staining, we investigated how the ionic strength of the buffer affects the equilibration process. Figure 2G–I show the intensity distributions at a dye:bp ratio of 1:5 at three different ionic strengths (0.05×, 0.5× and 5× TBE) without heating of the sample. The intensity distribution is more homogeneous at high ionic strengths, which could be explained in terms of the dependence of the dissociation rate constant of YOYO on ionic strength. At low ionic strengths (Figure 2G), the negative charges of the DNA are less screened, and the dissociation rate constant will be lower (29), meaning that the rate at which the YOYO-molecules rearrange to give a homogeneous staining of all DNA molecules in the sample is slower. At high ionic strengths, the dissociation rate will increase, which will accelerate the process of YOYO spreading evenly among the DNA molecules (Figure 2I).

### Heterogeneous staining as a tool

Although a homogeneously stained sample is important for several studies—both in bulk and on single DNA molecules—we here demonstrate how we can take advantage of the fact that our samples contain a wide distribution of binding ratios, where every single DNA-YOYO complex is unique. A similar approach has for example been explored by Christensen *et al.* (30) in fluorescence microscopy studies of single liposomes in a sample with a heterogeneous size distribution. By directly correlating the measured emission intensity to the size of each individual liposome, they were able to detect and understand size-dependent phenomena from a single sample. In analogy, heterogeneous staining of DNA can provide information about a broad spectrum of dye:bp ratios in a single sample, from the lowest amount of dye loading that can be visualized to a DNA that is fully saturated with YOYO.

**Figure 3.**
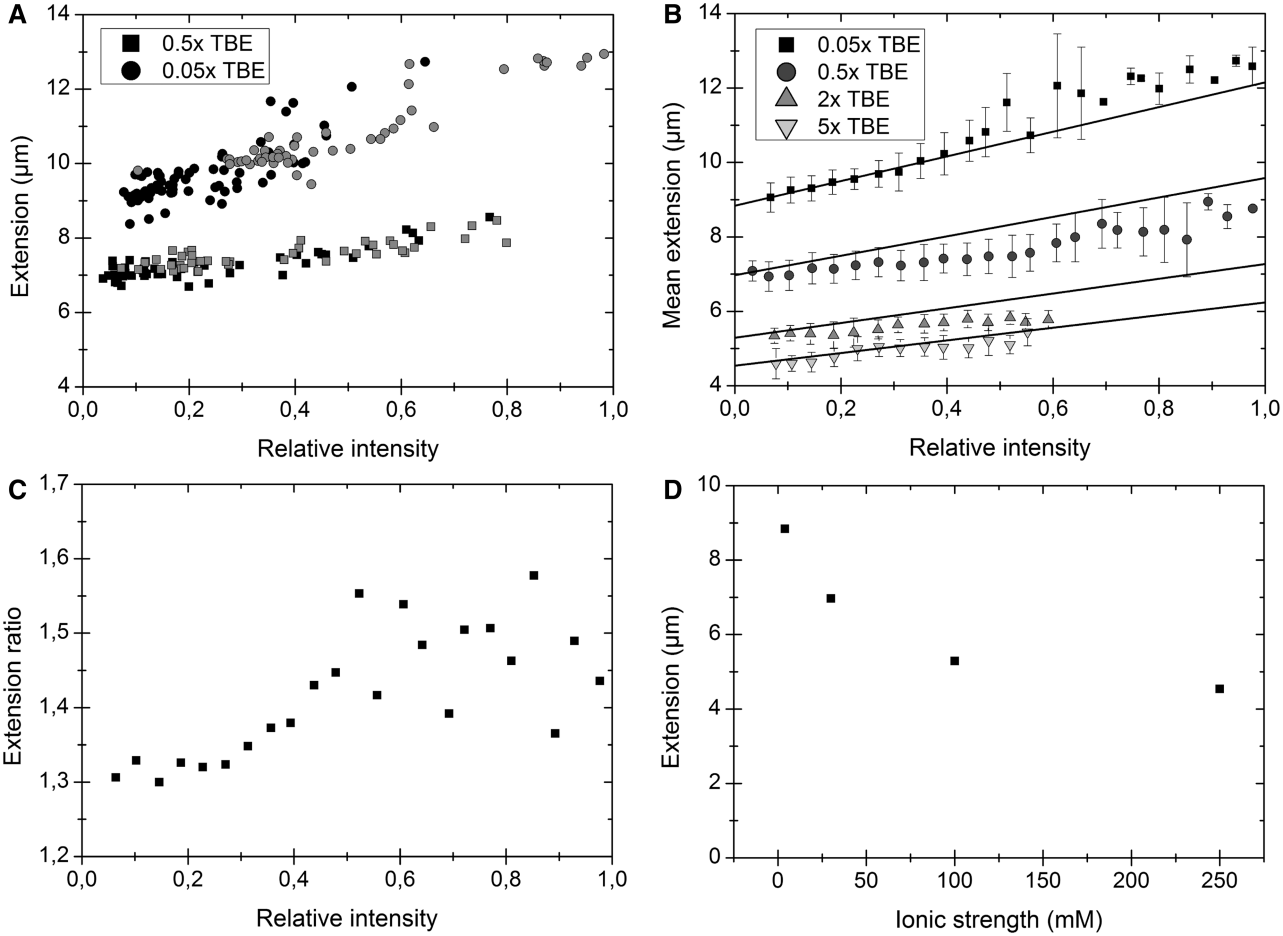
(**A**) Extension versus intensity for λ-DNA molecules in 0.05× TBE (circles) and 0.5× TBE (squares) at two different dye:bp ratios; 1:20 (black) and 1:5 (gray). (**B**) Extension and intensity distributions for ∼2650 λ-DNA molecules (heated and non-heated) at four different ionic strengths. The samples are sorted and binned according to relative intensity in steps of 0.04. The solid lines correspond to the expected increase in extension if the contour length of a fully intercalated DNA (1 dye per 4 bp) is extended with 0.51 nm per YOYO molecule and no other effects are considered (21,22). (**C**) Extension ratio, i.e. the extension at 0.05× TBE divided by the extension at 0.5× TBE, at different degrees of YOYO binding. (**D**) The extracted extension (from B) for native DNA at four different ionic strengths, converted from TBE according to ref (14).

Figure 3A shows the extension of λ-DNA plotted versus the fluorescence intensity for molecules from non-heated samples in 0.05× TBE and 0.5× TBE stained with dye:bp ratios of 1:20 and 1:5, respectively. All samples contain a wide range of dye:bp ratios, and the DNA extension increases as more dye is intercalated. Importantly, molecules from different samples at a particular ionic strength fall on the same curve, meaning that DNA molecules with specific emission intensities are equally extended and thus have the same dye loading, irrespective of the mean concentration of dye in the sample solution. It is thus possible to obtain information about all detectable dye:bp ratios (∼1:100 to 1:4) from a single sample instead of the time-consuming procedure of mixing and examining each dye concentration separately. Already in Figure 3A, based on only four experiments, it seems that the slopes of the two resulting curves differ, suggesting that YOYO affects the DNA extension differently at the two ionic strengths. This will be discussed in detail later in the text. We make the assumption that the emission of YOYO varies linearly with the amount of dye bound up to a dye:bp ratio of 1:4. This assumption is supported by earlier reports where YOYO shows a small dependence of quantum yield on binding density (31) and is explained by the two chromophores of YOYO always being in close proximity to each other, reducing the effect of energy transfer between two different YOYO molecules at high binding densities. As the environment in the intercalation pocket should be insensitive to changes in the ionic atmosphere around the DNA, we assume that the emission quantum yield of YOYO is similar at 0.05× and 0.5× TBE. This assumption is justified by the fact that the maximum emission intensity observed is almost identical at 0.05× and 0.5× TBE.

Next, we investigate how the extension of DNA in nanochannels varies with increased dye loading at different ionic strengths. Figure 3B shows a plot of the extension versus the intensity for YOYO labeled λ-DNA from both heated and non-heated samples at four different ionic strengths; 0.05×, 0.5×, 2× and 5× TBE. A number of interesting observations can be made: First, DNA is more extended at lower ionic strengths, which is in agreement with earlier studies, even though there is a controversy in the literature about the detailed underlying mechanisms (14,15,32). Second, we note that at higher ionic strengths (2× and 5× TBE), the maximum fluorescence intensity observed is almost halved compared with the maximum intensity in the samples at lower ionic strengths. We propose that a main contribution to this is the lower binding constant of polyvalent cations, such as YOYO, at higher ionic strengths (33), but small changes in emission quantum yield could potentially also contribute. Third, we observe that the changes in the extension of DNA as the binding ratio of YOYO increases are dissimilar for the ionic strengths investigated. The intercalative binding of YOYO leads to an increase in contour length of the DNA. Exactly how large this increase is has been debated, but two recent articles suggest an extension of 0.51 nm per YOYO molecule (22) or an increase in contour of 36% for fully saturated DNA (21), respectively, in good agreement with each other. The solid lines in Figure 3B show the expected increase in extension using the linearly extrapolated extension of native DNA as a starting point (discussed later in the text) and making the simplistic assumption that the increase in extension is solely due to an increase in contour length (21,22). Although this simple approximation fits fairly well with the observed change in extension at the lowest ionic strength (0.05× TBE), especially at low YOYO loading, the relative increase in extension is smaller at higher ionic strengths. Figure 3C shows a comparison of the extension at each intensity unit for molecules in 0.05× and 0.5× TBE. It is evident that the ratio between the extensions at the two ionic strengths is not constant but increases with increased dye loading. The consequence of YOYO binding to DNA, besides the mere extension due to intercalation, is that its four positive charges will be firmly bound to the DNA molecule. A less negatively charged YOYO–DNA complex should result in an extension that is smaller than expected from intercalation events only, due to a lower degree of repulsion from the channel walls as well as decreased intramolecular electrostatic repulsion. According to our observations, the reduction of the overall charge of the DNA, as more YOYO is intercalated, seems to have a greater influence at higher ionic strengths. We speculate that replacing four monovalent ions in the ionic atmosphere of DNA with four strongly bound charges on YOYO has a larger effect on the persistence length and/or the effective width at high ionic strength. This could potentially be due to the tighter binding of monovalent ions to DNA at low ionic strengths. Some studies suggest that the borate ions in the TBE buffer used affect the DNA (34). Negatively charged borate ions should however lead to a more extended DNA, rather than the opposite effect observed here.

Importantly, our assay also allows us to determine the extension of DNA without any dye bound by extrapolating the data to an emission intensity of zero (see ‘Materials and Methods’ section for details). Figure 3D shows the resulting extension of native λ-DNA at the different ionic strengths investigated. The extension of the DNA approximately doubles when going from high to very low ionic strength, in qualitative agreement with studies of YOYO stained DNA (14,15). The ability to extract information about DNA without dye bound is of extra importance based on our results above where we conclude that YOYO affects the DNA extension differently at different ionic strengths.

To conclude, we have demonstrated and characterized the heterogeneous staining of DNA by the commonly used dye YOYO using nanofluidic channels and fluorescence microscopy. Samples at standard conditions display a large heterogeneity in YOYO binding, but increased temperature and high ionic strength accelerates the equilibration. We conclude that electrostatic interactions are a key component in explaining the slow equilibration kinetics. The heterogeneous staining can be used in studies on how YOYO affects the physical properties of DNA. We demonstrate that the relative increase in extension of nanoconfined DNA as YOYO intercalates is larger at low ionic strengths and extract the extension of native DNA. Detailed exploration of how cationic intercalators affect DNA is of interest for the development of novel DNA-targeting drugs with improved functionalities. Furthermore, the use of heterogeneous staining has potential to be a fast and versatile tool for improving the statistics in single DNA molecule studies in general.

## FUNDING

Project funded by the Chalmers Area of Advance in Nanoscience and Nanotechnology. Funding for open access charge: Chalmers Area of Advance in Nanoscience and Nanotechnology.

*Conflict of interest statement*. None declared.
